# Family-Based Mindful Eating Intervention in Adolescents with Obesity: A Pilot Randomized Clinical Trial

**DOI:** 10.3390/children5070093

**Published:** 2018-07-06

**Authors:** Seema Kumar, Ivana T. Croghan, Bridget K. Biggs, Katrina Croghan, Rose Prissel, Debbie Fuehrer, Bonnie Donelan-Dunlap, Amit Sood

**Affiliations:** 1 Division of Pediatric Endocrinology, Department of Pediatric and Adolescent Medicine, Mayo Clinic, Rochester, MN 55905, USA; 2 Primary Care Internal Medicine, Mayo Clinic, Rochester, MN 55905, USA; croghan.ivana@mayo.edu (I.T.C.); DonelanDunlap.bonnie@mayo.edu (B.D.-D.); 3 Department of Psychiatry and Psychology, Mayo Clinic, Rochester, MN 55905, USA; biggs.bridget@mayo.edu; 4 Cancer Center, Mayo Clinic, Rochester, MN 55905, USA; croghan.katrina@mayo.edu; 5 Endocrinology/Nutrition, Mayo Clinic, Rochester, MN 55905, USA; prissel.rose@mayo.edu; 6 General Internal Medicine, Mayo Clinic, Rochester, MN 55905, USA; fuehrer.debbie@mayo.edu (D.F.); sood.amit@mayo.edu (A.S.)

**Keywords:** mindful eating, obesity, adolescents

## Abstract

Mindfulness has gained attention in the treatment of obesity. However, there is a paucity of data on family-based training in mindful eating in children. The objective of this pilot randomized clinical trial was to evaluate the feasibility and acceptability of a family-based mindful eating intervention (MEI) in adolescents with obesity, and to compare the efficacy of the MEI versus standard dietary counseling (SDC) for decreasing weight and improving cardiometabolic risk markers. Twenty-two adolescents (age 14.5–17.9 years) and parent pairs were randomized to the MEI or SDC. The MEI was administered in four 90-min sessions over 10 weeks and SDC was provided at baseline, 12 weeks, and 24 weeks. Despite the requirement of more frequent visits with the MEI, adolescents and parents attended 100% of the sessions and there were no dropouts in that group. High density lipoprotein (HDL) cholesterol increased in the SDC group, but not in the MEI group. Adolescents receiving the MEI demonstrated an increase in awareness at 24 weeks (*p* = 0.01) and a decrease in distraction during eating at 12 weeks (*p* = 0.04), when compared with the SDC group. The family-based MEI showed feasibility and acceptability in adolescents with obesity. Future studies with more intense therapy and larger sample sizes are warranted to examine the role of mindful eating in treating pediatric obesity.

## 1. Background

The problem of childhood obesity has reached epidemic proportions [1]. Currently, 18.5% of children in the United States have obesity and 6% have severe obesity [1]. Childhood obesity is associated with several adverse health consequences, including type 2 diabetes, dyslipidemia, and hypertension [2,3,4]. If established by adolescence, obesity, and its associated health problems, is likely to persist into adulthood [2]. While lifestyle modification is considered the cornerstone of pediatric obesity treatment, the efficacy of behavioral modification programs to address obesity through the promotion of healthier eating and an active lifestyle is modest and is limited by dropout rates of 27–73% [5,6,7,8,9]. Additionally, there is limited evidence for the long-term sustainability of weight loss in children with obesity [10,11,12]. Therefore, additional strategies are warranted to enhance the efficacy and sustainability of lifestyle modifications, including dietary habits, in the management of childhood obesity.

Mindfulness is defined as “the awareness that arises from paying attention on purpose, in the present moment and non-judgmentally” [13]. Eating behaviors play an important role in excess weight gain. Mindfulness has gained attention as an avenue for the treatment of obesity through modification of problematic eating behaviors, such as excess caloric intake in response to external cues (e.g., portion size) [14,15,16,17]. Studies investigating the effects of mindfulness on obesity-related eating behaviors have focused almost exclusively on adult samples, with only two including late-adolescent/young adult participants [15]. Several studies have demonstrated the benefits of mindfulness-based weight loss intervention for adults [18,19]. Mindfulness-based interventions have demonstrated effectiveness in improving binge eating, emotional eating, and external eating behaviors [15], as well as improving self-control [20]. Mindfulness techniques have been adapted for use with adolescents and children as young as preschool age, with at least 15 reported studies supporting their feasibility and acceptability when taught to children or to children and parents together [21,22]. The limited research suggests mindfulness can improve adolescents’ self-regulation, particularly in response to stress [23]. However, there is a paucity of data on the effect of family-based mindful eating interventions (MEI) in the management of pediatric obesity or in young people’s eating behaviors [24].

The objectives of this pilot randomized clinical trial were to (i) evaluate the feasibility and acceptability of a family-based MEI in adolescents with obesity and (ii) compare the efficacy of a family-based MEI versus standard dietary counseling (SDC) for decreasing weight and improving cardiometabolic risk markers in adolescents with obesity.

## 2. Subjects and Methods

### 2.1. Design Overview

This was a two-arm, parallel-group, pilot, randomized, blinded (outcome assessor and statistician), dietary counseling group controlled clinical trial in adolescents with obesity. The trial was registered on ClinicalTrials.gov (NCT01764113), and was approved by the Mayo Clinic Institutional Review Board (12-006349).

### 2.2. Setting and Participants

Adolescents between the ages of 14–17 years were considered eligible if their body mass index (BMI) was at or above the 95th percentile for age and gender. Age and gender-specific BMI percentiles and *z*-scores were calculated using the standards recommended by the Centers for Disease Control and Prevention [25].

Exclusion criteria included: (i) currently attending a supervised weight loss program; (ii) underlying genetic or endocrine cause for weight gain; (iii) type 1 or type 2 diabetes mellitus; (iv) ongoing treatment with receiving insulin, metformin, or oral hypoglycemic medications; (v) use of oral glucocorticoids in the previous two months; (vi) current cancer; (vii) established diagnosis of psychiatric illness in the previous six months; (viii) inability of the participant or parent to provide informed assent/consent or inability of the same parent to attend all of the intervention sessions and (ix) study visits along with their adolescent. 

Participants were recruited from primary care practices (general pediatrics and family medicine) and pediatric specialties at the Mayo Clinic, Rochester, MN, USA.

### 2.3. Randomization and Interventions

Participants and one parent were randomized in a 1:1 ratio to the MEI group or SDC. Randomization was performed using a computer-generated randomization schedule. The same parent was required to attend the intervention sessions, as well as the study visits, in either group throughout the duration of the study.

Informed assent was obtained from the adolescents and informed consent was obtained from the parent who had enrolled in the study. Interventions for both the arms were group based (3–4 adolescent and parent pairs per group). The sessions in both the MEI and SDC treatment arms were conducted in an outpatient clinic setting on a weekday between 6 p.m. and 7:30 p.m. A healthy meal consisting of a sub sandwich with low fat meats and veggies with fruit, baked chips, and zero calorie lemonade was served before the meetings at 5:30 p.m.

The mindful eating program was administered over four 90-min sessions (baseline, 1 week, 6 weeks, and 10 weeks) by a team comprised of a physician and a trained mind-body therapist.

The adolescents and parents in the MEI arm attended concurrent sessions (one for adolescents and the other one for parents only) held in different rooms and met each other at the end of the session only. The program had two components: an approach toward mindfulness, and an application of the mindfulness principles to eating. The first session emphasized mindfulness and stress management strategies, including brief practices to improve attention and bring greater gratitude and compassion in life. In the next three sessions, participants learned mindful eating principles, including learning to be more cognizant of what one is eating, attuning with one’s body to assess hunger and appropriate size of serving, cultivating gratitude for food and its preparers, relaxing for a moment prior to eating, paying purposeful attention to the color and aroma of food, eating slowly in small bites, enjoying each bite fully, and paying attention to the sensation of fullness from the stomach. Participants were also taught skills to avoid automatic eating, develop better self-control when offered appetitive calorie dense foods, and shopping with better awareness of the calorie density and nutritive value of foods. Each session offered a combination of a scientific perspective combined with stories and specific skills in mindfulness and mindful eating.

Adolescent/parent pairs in the SDC arm received three 90-min sessions of dietary counseling (parents and adolescents together) by a registered pediatric dietician at baseline, 12 weeks, and 24 weeks. The dietary counseling in the SDC group focused on portion control, decreasing intakes of calorie dense fast food/convenience store foods, and substituting with healthier foods consistent with routine care for youth with obesity. Throughout each session, the focus was on further educating teens and their caregiver through discussion and utilization of educational tools, such as using the provided meal to discuss portion control, satiety, energy density, and the nutrients provided in the food. In addition, using meaningful food labels, food models, and various plate, cup, and bowl sizes to engage thought provoking discussion were used along with the didactic means of educating teens and their care provider.

### 2.4. Outcomes and Follow Up

Participants were evaluated by study staff at baseline, 12 weeks, and 24 weeks. The primary outcomes measures were change in weight, BMI, and BMI *z*-score. The secondary outcomes measures were change in fasting glucose, insulin, lipids, high sensitivity C-reactive protein (hs-CRP), and domains in the mindful eating questionnaire (MEQ) and weight-efficacy lifestyle questionnaire (WEL).

Weight, height, and blood pressure were obtained at baseline, 12 weeks, and 24 weeks, and were collected by trained research assistants using standardized instruments. Blood glucose, insulin, total cholesterol, high density lipoprotein (HDL) cholesterol, triglycerides, and hs-CRP were obtained after a 10–12 h overnight fast at baseline and at 24 weeks. Plasma glucose was measured by hexokinase enzymatic assay, and serum insulin was measured using commercial electrochemiluminescence immunoassay kits. Total cholesterol, HDL cholesterol, and triglyceride levels were measured by an enzymatic calorimetric assay. Non-HDL cholesterol was calculated as total cholesterol minus HDL cholesterol. Low density lipoprotein (LDL) cholesterol was calculated using the equation: LDL = total cholesterol − HDL cholesterol − (triglycerides/5). Measurement of hs-CRP was performed using particle-enhanced immunonephelometry (Siemens Healthcare Diagnostics, Deerfield, IL, USA).

The MEQ [26] and WEL [27] were administered at baseline, 12, and 24 weeks. The MEQ is a 28-item scale designed to assess non-judgmental awareness of physical and emotional cues associated with eating. For each item, respondents rate the frequency of the listed behavior and items are scored 1–4, with 4 indicating higher mindfulness. Resulting scores include a summary score and five subscales: disinhibition, awareness, external cues, emotional response, and distraction. The WEL is a 20-item eating self-efficacy scale consisting of a total score and five situational factors: negative emotions, availability, social pressure, physical discomfort, and positive activities. For each item, respondents rate their level of confidence in their ability to resist eating in the given situation on a 10-point scale, from 0—“not confident”, to 9—“very confident”.

### 2.5. Sample Size Calculation and Statistical Analysis

A priori power analysis conducted using G*Power 3 [28] indicated that a sample size of 40 participants (20 for each arm) would be required to detect the medium to large effect sizes observed in similar previous research assuming 0.80 power with a two-tailed test and *α* set at 0.05. Since the trial was of a pilot nature and the primary aim was to examine the feasibility and acceptability of the MEI, recruitment was discontinued at the end of the trial despite a total enrollment of only 22 participants. 

Continuous variables were summarized with mean ± standard deviation (SD), or median and interquartile range (IQR), as appropriate. Categorical variables were summarized as a frequency and percentage. Differences between time points were assessed within each study group with Wilcoxon signed-rank tests, and the paired differences were compared between study groups with Wilcoxon rank-sum tests. *p*-Values less than 0.05 were considered statistically significant. All analyses were performed using SAS version 9.3 (SAS Institute Inc., Cary, NC, USA) [29]. Subgroup analyses were not performed due to the small sample size.

## 3. Results

**Figure 1:**
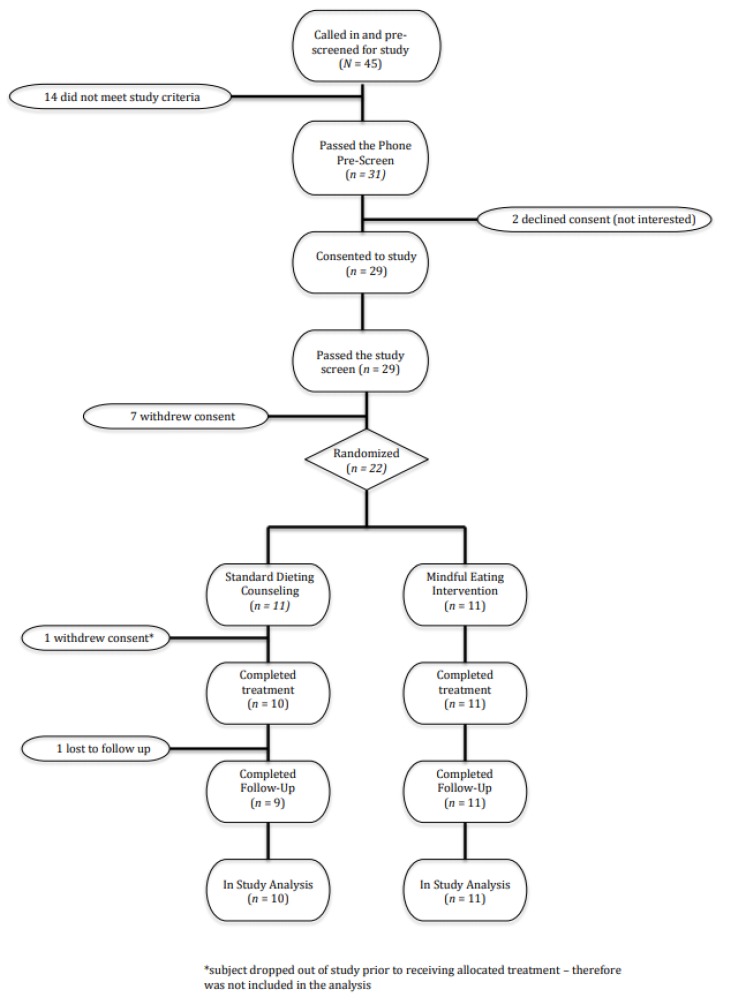
Study flow.

A total of 22 adolescents (age range 14.5–17.9 years) were enrolled in the study (Figure 1). Out of 45 adolescents that were called in and prescreened for the study, 31 passed the phone screen and 14 did not meet the study criteria (Figure 1). Twenty-nine adolescents consented to the study and two declined. Seven out of the 29 consented adolescents withdrew consent and; therefore, 22 adolescents were randomized to either of the study arms (11 in each group). The study was conducted between 7 December 2012 and 19 December 2013.The interventions were conducted at two different times of the year due to the convenience in the recruitment timing. The first group of 14 participants (8 males, 6 females) began intervention in winter (February 2013) and these were equally divided between the MEI and SDC (7 in each group). The remaining 8 patients (4 males, 4 females) began receiving the intervention in June 2013 and these were also equally split between the two intervention groups (4 in each group). 

Anthropometric and laboratory characteristics of the participants are detailed in Table 1.

Age, gender, and race were not different between the two groups (*p* > 0.05). The median age (quartile, Q1, Q3) was 15.6 (15.2, 16.8) years in the SDC group and 17.1 (15.5, 17.4) years in the MEI group. There were 12 male and 10 female participants, with 7/11 (63.6%) participants in the SDC group (63.6%) and 5/11 (45.5%) participants in the MEI group were male. Nineteen out of 22 participants were white and three were non-white (one in the SDC group and two in the MEI group).

Baseline BMI was not significantly different between the two groups (median (Q1, Q3): 32.9 (30.9, 36.2) kg/m^2^ and 34.9 (32, 36.6) kg/m^2^ in the SDC and MEI group, respectively; *p* > 0.05). Baseline BMI *z*-score also was not different between the two groups (median (Q1, Q3): 2.4 (1.9, 3.2) and 2.5 (2.3, 3.0) in the SDC and MEI group, respectively; *p* > 0.05). There were no differences in the laboratory parameters between the two groups (*p* > 0.05, Table 1).

All 11 adolescent and parents in the MEI group attended all four intervention sessions, as well as the study visits, at 12 weeks and 24 weeks. Therefore, there were no drop outs in the MEI group. One participant in the SDC group dropped out after the first session and did not return for the 12-week or 24-week visit. Data from 10 participants in the SDC group and from 11 participants in the MEI group were included in the analysis.

Body mass index and BMI *z*-score in the MEI group were higher at 12 weeks and 24 weeks (*p* < 0.05) relative to baseline (Table 2). Body mass index and BMI *z*-scores in the SDC group were higher (*p* < 0.05) at 24 weeks compared to baseline (Table 2). However, there were no significant differences in the change in weight, BMI, and BMI *z*-score from baseline to 12-week or 24-week time points between the two groups (*p* > 0.05 for all, Table 2).

There was a statistically significant difference in the δ HDL cholesterol between the SDC and MEI groups, with HDL cholesterol increasing in the SDC group (median (Q1, Q3): 7 (2, 10) mg/dL), but not in the MEI group ((−1 (−2, 3) mg/dL); *p* = 0.0245). There was no difference in changes in other laboratory measures between the two groups.

No adverse side effects were reported by study participants in either of the groups.

Adolescents in the MEI group demonstrated an increase in awareness at 24 weeks and a decrease in distraction during eating at 12 weeks relative to the SDC group (*p* = 0.01 and *p* = 0.04, respectively, Table 3).

## 4. Discussion

We demonstrate the feasibility and acceptability of a family-based mindful eating intervention in adolescents with obesity. To our knowledge, this is the first randomized clinical trial to test the feasibility, acceptability, and short term weight loss efficacy of a family-based mindful eating intervention in adolescents with obesity.

Despite the need for four 90-min sessions for adolescents and their parents over a period of 10 weeks, we noted 100% attendance in the four training sessions by adolescents and their parents. In addition, all participants in the MEI group returned for assessments at 12 weeks and 24 weeks. This level of attendance in the intervention sessions and retention over the 24-week period is remarkable given reported dropout rates in behavioral treatments for pediatric obesity [9], and the scant data on the feasibility and acceptability of a mindful eating-based intervention in children and adolescents with obesity [30]. In contrast to our findings, Daly and colleagues reported only 57% retention in a randomized feasibility study aimed at weight loss in adolescent Latino females [30]. The mindful eating intervention in that study was administered to adolescents recruited in a public high school in southwestern United States and was not delivered to parents. The differences in retention rates may be related to differences in the ethnicity and gender distribution of participants as the majority of our study participants were white, and males comprised more than half of our study population. Additionally, there were several differences in the specifics of the mindful eating intervention, such as the frequency of the intervention sessions (90-min session every week for six weeks in the study by Daly versus four 90-min sessions over a period of 10 weeks in our study), time of the day (afterschool setting versus evening time in our study), and setting (classroom versus outpatient clinic in our study). The mindful eating intervention in our study, on the other hand, was conducted in the late evening with an evening meal provided to all participants and their parents. Finally, involvement of the family, as was required in our study, could have also contributed to a greater retention. Our finding of a higher retention in the MEI group is all the more remarkable in view of the longer follow up period in our study (24 weeks versus 10 weeks in the study by Daly and colleagues [30]).

The MEI was not efficacious in decreasing BMI or BMI *z*-score at 12 weeks or 24 weeks in our study. Participants in both groups had an increase in BMI and BMI *z*-scores at 24 weeks. These findings are not surprising given the modest efficacy of behavior modifications on severity of adiposity [31]. Similar to our study, overweight/obese adolescent females in another study by Shomaker and colleagues also exhibited an increase in BMI despite receiving mindfulness-based group intervention [32]. In contrast, a 1.4 kg/m^2^ decrease in BMI was noted at 10 weeks in Latino adolescent females receiving MEI, but no subsequent BMI measurements were available at 24 weeks in their study [30]. The greater frequency of MEI in the study in Latino adolescent females (six weekly 90-min sessions) compared to our study (four 90-min sessions over a period of 10 weeks) may partly account for differences in the effects on BMI. Mindful eating-based interventions have been shown to result in weight loss among children with Prader-Willi syndrome [33,34,35]. Several studies in adults have also demonstrated modest benefits in adults [18,19,36], with a mean weight loss of 4 kg and a mean decrease in BMI of 1.3 kg/m^2^ in one study [19]. The lack of improvement in weight or BMI in the participants in our MEI or SDS groups may be due to the low intensity of the intervention, with only 6 h of contact over a period of six months, despite the most recent The United States Preventive Task Force guidelines recommending >26 h “over a period of 2–12 months” [37]. It remains unknown if an addition of a greater number of hours of mindfulness-based intervention would lead to an improvement in outcomes of adolescents with obesity.

We noted improvements in awareness and in reduced distraction during eating among the adolescents receiving the mindful eating intervention. No differences in mindfulness awareness were noted in adolescent Latino females receiving a mindful eating intervention [30]. Unfortunately, changes in these domains did not result in an improved weight status during the study period. The inclusion of dietary assessments in future studies would further the understanding of whether changes in these mindful eating domains translate into changes in dietary quality or caloric intake that could, over time, lead to changes in weight status.

The strengths of our study include the randomized clinical trial design with the presence of a control group receiving standard dietary counseling on a low fat, portion controlled dietary plan. We had excellent retention with follow up on all but one out of the 22 participants at six months from the time of enrollment.

The most significant limitation of our study is its pilot nature and small sample size. The study was underpowered to determine the efficacy of the MEI on weight and cardiometabolic risk factors in adolescents with obesity. Another limitation was the low intensity of the intervention, with the total number of hours of contact of 6 h being significantly below the >26 h “over a period of 2–12 months” recently recommended by national experts [37]. The duration of follow up was short and, therefore, outcomes beyond six months could not be assessed. Other important limitations were the lack of information regarding pubertal staging and body composition, and lack of data on disordered eating patterns, such as binge eating and emotional eating, as well as on quality/quantity of dietary intake and physical activity. Finally, the study findings cannot be generalized due to a lack of ethnic and racial diversity among the participants.

## 5. Conclusions

A family-based mindful eating intervention was found to be feasible and acceptable in adolescents with obesity. No beneficial changes in weight, BMI, or any of the cardiometabolic risk factors were observed. Further studies with a greater intensity of interventions and larger sample size are warranted to examine the role of mindful eating interventions in the treatment of childhood obesity.

## Figures and Tables

**Table 1:** Anthropometric and laboratory characteristics of study participants.

	Standard Dietary Counseling (*n* = 11)		Mindful Eating Intervention (*n* = 11)		Difference between the Two Arms
	Median	Q1, Q3	Median	Q1, Q3	*p-*Value
Age (years)	15.6	15.2, 16.8	17.1	15.5, 17.4	NS
Weight (kg)	103.7	87, 123	98.7	95.9, 106.4	NS
BMI (kg/m^2^)	32.9	30.9, 36.2	34.9	32, 36.6	NS
BMI *z*-score	2.4	1.9, 3.2	2.5	2.3, 3.0	NS
Glucose (mg/dL)	88	81, 96	92	88, 99	NS
Insulin (mcIU/mL)	21.8	17.7, 24.5	29.3	15.6, 35.1	NS
Total cholesterol (mg/dL)	176	133, 183	144	124, 180	NS
HDL cholesterol (mg/dL)	41	33, 53	35	31, 48	NS
LDL cholesterol (mg/dL)	101	69, 106	81	77, 112	NS
Non-HDL cholesterol (mg/dL)	126	91, 146	108	93, 132	NS
Triglycerides (mg/dL)	174	81, 219	104	80, 134	NS
hs-CRP (mg/L)	1.7	0.5, 4.3	3.2	0.4, 5.7	NS

NS, *p* > 0.05. BMI, body mass index; HDL, high density lipoprotein; LDL, low density lipoprotein; hs-CRP, high sensitivity C-reactive protein; Q, quartile.

**Table 2:** Change in anthropometric and laboratory measures during follow up.

		Standard Dietary Counseling (*n* = 10)		Mindful Eating Intervention (*n* = 11)		Difference between the Two Groups
Parameter	Time	Median	Q1, Q3	Median	Q1, Q3	*p*-Value
Weight (kg)	Week 12	−0.1	−1.4, 1.2	2.3	−0.7, 3.2	NS
	Week 24	2.5	1.5, 3.3	4.0	−0.2, 5.2	NS
BMI (kg/m^2^)	Week 12	−0.3	−0.9, −0.1	0.6	−0.2, 1.2	NS
	Week 24	1	0.4, 1.1	0.9	0.2, 2.3	NS
BMI *z*-score	Week 12	−0.1	−0.2, 0	0.1	0, 0.2	NS
	Week 24	0.2	0.1, 0.2	0.2	0, 0.4	NS
Glucose (mg/dL)	Week 24	2	−3, 6	−4	−8, 6	NS
Insulin (mcIU/mL)	Week 24	3.5	1.4, 6.6	0.3	−4.5, 4.8	NS
Total cholesterol (mg/dL)	Week 24	14	4, 24	3	−11, 28	NS
LDL cholesterol (mg/dL)	Week 24	1	−7, 12	8	−11, 20	NS
HDL cholesterol (mg/dL)	Week 24	7	2, 10	−1	−2, 3	0.0245
Triglycerides (mg/dL)	Week 24	9	2, 27	7	−8, 24	NS
hs-CRP (mg/L)	Week 24	0.3	0.2, 0.4	0.2	−1.4, 1.2	NS

NS, *p* > 0.05.

**Table 3:** Changes in mindful eating questionnaire (MEQ) and weight-efficiency lifestyle questionnaire (WEL) domains.

	Standard Dietary Counseling (SDC) (*n* = 10)			Mindless Eating Intervention (MEI) (*n* = 11)		
	Baseline ^a^	Week 12 ^a^	Week 24 ^a^	Baseline ^a^	Week 12 ^a^	Week 24 ^a^
Mindful eating questionnaire						
Awareness	2.5 (0.6)	2.5 (0.7)	2.4 (0.7)	2.1 (0.5)	2.4 (0.4)	2.5 (0.5) *
Disinhibition	2.7 (0.7)	2.7 (0.6)	2.6 (0.8)	3.0 (0.4)	2.9 (0.3)	2.9 (0.4)
Distraction	3.7 (0.3)	3.6 (0.3)	3.7 (0.5)	3.8 (0.2)	3.4 (0.4) **	3.6 (0.4)
Emotional	2.6 (0.3)	3.1 (0.4)	2.9 (0.3)	2.9 (0.4)	2.8 (0.5)	2.9 (0.3)
Weight efficacy lifestyle questionnaire						
Total score	6.2 (2.0)	6.5 (1.9)	6.5 (2.0)	7.0 (1.2)	6.5 (1.2)	7.0 (1.1)
Negative emotions	6.5 (2.5)	6.4 (2.8)	6.9 (2.4)	6.8 (2.2)	6.7 (1.7)	7.0 (1.6)
Availability	5.2 (2.2)	5.7 (2.3)	5.6 (2.2)	5.9 (1.5)	5.4 (1.9)	6.1 (1.4)
Social pressure	5.8 (2.7)	6.3 (2.4)	5.7 (2.0)	7.0 (1.2)	6.2 (1.7)	6.8 (1.5)
Physical discomfort	6.9 (1.8)	7.3 (1.6)	7.4 (1.9)	7.7 (1.2)	7.3 (1.3)	7.7 (1.4)
Positive activities	6.7 (1.8)	7.0 (1.7)	6.8 (2.1)	7.4 (1.1)	7.0 (0.8)	(0.9)

^a^ Data are presented as mean ± SD (standard deviation); difference between MEI and SDC groups with regards to change from baseline: * MEQ awareness (week 24-baseline difference), *p* = 0.01; ** MEQ distraction (week 12-baseline difference), *p* = 0.04.
